# Successful and Safe Treatment of Chronic Spontaneous Urticaria with Omalizumab in a Woman during Two Consecutive Pregnancies

**DOI:** 10.1155/2015/368053

**Published:** 2015-01-29

**Authors:** Misbah Nasheela Ghazanfar, Simon Francis Thomsen

**Affiliations:** Department of Dermatology, Bispebjerg Hospital, 2400 Copenhagen NV, Denmark

## Abstract

Chronic spontaneous urticaria is an itching skin disease characterised by wheals, angioedema, or both present for more than six weeks. Omalizumab is a humanized anti-IgE monoclonal antibody recently approved for treatment of chronic urticaria. Several randomised controlled trials have investigated the safety, tolerability, and efficacy of omalizumab for chronic urticaria. The safety of omalizumab in pregnancy is not known. We describe a female patient with chronic spontaneous urticaria who was treated with omalizumab continuously through two consecutive pregnancies with convincing results and no apparent toxicity.

Urticaria is a severely itching skin disease characterised by wheals, angioedema, or both. It is divided into acute (present < 6 weeks) or chronic (present > 6 weeks) types [1]. Chronic urticaria is further subdivided into chronic spontaneous urticaria (CSU) and chronic inducible urticaria (CINDU), the latter appearing repeatedly after physical stimuli such as heat, cold, or sun exposure [2].

International guidelines recommend nonsedating antihistamines once daily as first-line therapy of CSU or CINDU; if no effect is observed it is recommended to increase the dose of antihistamine up to fourfold. If still refractory, third-line options include add-on therapy of omalizumab, ciclosporin, or montelukast [2].

Omalizumab is a humanized anti-IgE monoclonal antibody recently approved for treatment of chronic urticaria [1, 2]. Several randomised controlled trials have investigated the safety, tolerability, and efficacy of omalizumab for chronic urticaria. Particularly, studies have shown that omalizumab not only reduces urticarial symptoms and activity markedly, but also reduces the need for additional medication and improves quality of life [2]. Collectively, clinical phase II and III studies [3–7] have concluded that the ideal omalizumab dose for maximum effect and tolerability is 300 mg once every four weeks. Overall, no significant difference in adverse effects has been observed between patients receiving different doses of omalizumab and patients receiving placebo [4, 6, 7].

Although omalizumab apparently is safe and tolerable, based on the observations from clinical trials, data on long-term safety are still lacking. Notably, the safety and tolerability of omalizumab used in chronic urticaria during pregnancy have not been investigated, one exception being a woman from Germany, in whom no side effects or foetotoxicity was observed [8]. We describe a female patient with CSU who was treated with omalizumab continuously through two consecutive pregnancies with convincing results and no apparent toxicity.

The patient was a 32-year-old woman with severe CSU with angioedema, atopic dermatitis, and asthma that was well controlled with terbutaline, who in August 2009 was referred to our department. At time of referral she also used the antidepressant escitalopram but did not require treatment for atopic dermatitis. The patient suffered from urticarial rashes primarily on the lower back, neck, dorsal side of hands, and the legs. She experienced symptoms of urticaria on a daily basis including particularly troublesome symptoms during night time that interfered with sleeping. A chest X-ray, urine and throat cultures, and routine blood tests including hepatitis B and C, tuberculosis, and helicobacter pylori were normal. Before treatment serum total IgE was 159 KIU/L (slightly elevated), whereas blood eosinophils were normal. She screened negative for antinuclear antibodies and a serum-induced basophil histamine release test was normal. For three months she was treated with high-dose nonsedating antihistamines (cetirizine 10 mg) up to three times daily but without effect. Hereafter, azathioprine 100 mg daily was added, and, after ten days she experienced a severe episode of facial angioedema that was treated with prednisolone for four days. She was then treated with an increased dose of 125 mg azathioprine in combination with nonsedating antihistamines four times daily due to the severe urticaria activity which could not be controlled alone on antihistamines.

After six months without significant symptomatic relief, she was switched to the TNF inhibitor adalimumab (40 mg s.c. once every two weeks) and an improvement was observed; however hives were still emerging at the end of the dosing intervals. In August 2010, the patient was therefore switched to omalizumab 150 mg s.c. every two weeks and she then experienced a rapid (within the first 24 hours) and complete resolution of symptoms without any side effects or injection site reactions of the treatment. After six months' treatment she had an unusual asthmatic episode three days after receiving omalizumab, which was treated with 37.5 mg prednisolone for 10 days. It was not possible to clarify the trigger. In May 2011, omalizumab was paused to see whether the disease was permanently in remission, but already in June 2011 omalizumab was resumed due to symptoms of urticaria. In August 2011 she experienced a short-term exacerbation of urticaria while she was treated in hospital for a peritonsillar abscess.

While still treated with omalizumab she became pregnant in November 2011, and, after having been thoroughly informed about the potential risks, she decided to continue treatment with omalizumab throughout pregnancy. In July 2012, she gave birth to a healthy girl with a birth weight of 4030 g and no congenital abnormalities. Due to prolonged pregnancy, labour was induced in week 42 of gestation. However, the child was delivered by acute Caesarean section due to slow progression of labour and failed vacuum extraction. The baby was not breastfed. Three years and two months after delivery the child is still healthy with no developmental abnormalities.

After delivery the patient continued with her regular dose of omalizumab until she became pregnant again. As she was now experiencing complete remission of urticaria, the dose of omalizumab was changed to 300 mg s.c. once monthly. Again, no complications or side effects were observed during her second pregnancy and in September 2014 she delivered, by elective Caesarean section after week 39 of gestation, another healthy girl with a birth weight of 3500 g and no congenital abnormalities. The baby was not breastfed. Five months after delivery the child is still healthy with no developmental abnormalities (Figure 1).

Clinical trials have shown that not only does omalizumab significantly reduce disease activity and symptoms of urticaria rapidly it is also well-tolerated and safe. Overall, the reported incidence of adverse effects is the same in patients treated with omalizumab compared to placebo-treated patients, although some studies [5, 7] did note that a higher dose of omalizumab increased the occurrence of adverse effects such as headache and injection side reactions, however, with no difference in serious adverse effects reported. The most common adverse effects were headache, diarrhea, nasopharyngitis, and respiratory tract infections [4, 7].

While the majority of the patients in the clinical phase studies were women [3–7] pregnant women still remain unexplored. Due to ethical reasons, it is not possible to include pregnant women in clinical trials where drug safety and mechanism of action are not well known. Omalizumab inhibits the binding of IgE to IgE receptors on mast cells and basophils, thereby reducing the activity of these cells [8]; however, the overall drug mechanisms of omalizumab in urticaria are not clarified. Therefore, it is difficult to exclude any harmful effects of omalizumab in pregnancy.

Only one previous case study (a woman suffering from three different types of urticaria: CSU, pressure urticaria, and symptomatic dermographism) suggests that omalizumab is safe and effective during pregnancy [9]. Furthermore, among 191 asthmatic women treated with one or more doses of omalizumab and registered in the Xolair Pregnancy Registry (EXPECT), no apparent increased birth prevalence of major anomalies was observed [10].

A limitation of our study is that the patient's urticaria activity was not followed prospectively with validated urticaria activity scores such as the UAS (Urticaria Activity Score), DLQI (Dermatology Life Quality Index), or UCT (Urticaria Control Test) [2]. These scoring systems would have provided a better basis for comparing urticaria activity across studies and during treatment with omalizumab.

Our report of successful and safe treatment of CSU with omalizumab during two consecutive pregnancies is important as many young women of childbearing potential suffer from urticaria. It is critical that an effective and safe treatment is provided for these patients even during pregnancy and lactation as urticaria has a substantial negative impact on quality of life.

## Figures and Tables

**Figure 1:**
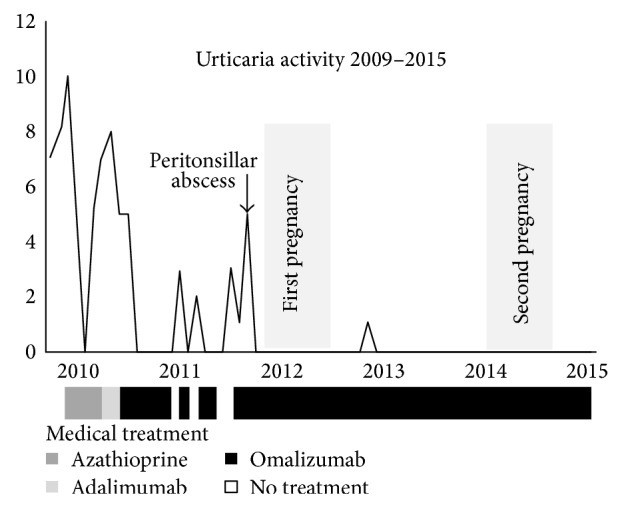
Urticaria activity from 2009 to 2015. Note: as no validated scoring system of urticaria activity was used during the course of treatment, the urticaria activity was scored on the basis of the severity of symptoms of remission or relapse described in the medical journal of the patient. The activity (*y*-axis) is scored from 0 (no urticarial activity) to 10 (most severe urticarial activity) depending on the description in the patient record. *x*-axis denotes course of treatment with graphical representation of periods with medical treatment.
